# Prevalence of HIV-2 and ART treatment coverage in Northern Sierra Leone

**DOI:** 10.1186/1471-2334-14-S2-P15

**Published:** 2014-05-23

**Authors:** Nell G Bond, Augustine Goba, Danielle Levy, Lina M Moses, Sallieu K Sesay, Idriss Bangura, Matthew Kemoh Gibateh, Sheik H Khan, Preston A Marx

**Affiliations:** 1 Tulane University School of Public Health and Tropical Medicine, New Orleans, Louisiana, USA; 2 Tulane University Lassa Fever Project, Kenema, Sierra Leone; 3 Tulane University School of Medicine, New Orleans, Louisiana, USA; 4 Kabala Government Hospital, Kabala, Sierra Leone; 5 Sierra Leone Ministry of Health and Sanitation, Freetown Sierra Leone; 6 Tulane University National Primate Research Center, Covington, Louisiana, USA

## 

Acquired immunodeficiency syndrome caused by HIV-1 or HIV-2, affects 35.3 million people worldwide. Nine HIV-2 subtypes originating from sooty mangabeys in West Africa have been described. Subtypes A and B are epidemic while C to I are crossovers that are known in single persons. HIV-2F is an exception among non-epidemic subtypes, being pathogenic and found in two persons, both from Northern Sierra Leone, suggesting transmissibility. Very little data are published concerning the distribution and prevalence of HIV2 in Northern Sierra Leone despite a new pathogenic HIV2 emerging from this region. Data on ART treatment coverage is also lacking.

Subjects presenting for voluntary HIV test and those referred by healthcare providers were enrolled following informed consent. This represents a targeted, higher risk population than the general population. Commercial HIV1/2 rapid tests were used in the field. PCR and NGS methods are being used to determine prevalence of newly emerging HIV-2F. A questionnaire was administered to collect demographic information and treatment history in those testing positive.

Currently the prevalence of HIV in the targeted sample population is 5.98%. Interestingly, when compared to the last published data on HIV-2 in the region, prevalence has increased by a factor of 32, from 0.021 to 0.68%. 77% of HIV positive persons were newly identified cases. Of those previously testing HIV positive, only 41% were currently on treatment compared to 61% ART coverage in HIV positive persons in low and middle-income countries globally.

Our data indicate the prevalence of HIV has increased in Sierra Leone since the civil war. Further data are needed to conclusively show prevalence changes of HIV in Northern Sierra Leone on a population level. The data also show that ART treatment rates in Northern Sierra Leone are significantly lower than the global average highlighting the need for improved case identification and treatment provision in this resource poor setting. Sequencing of HIV positive samples to determine the subtype is in process. This study provides a basis for further population based study of the HIV strains circulating in Northern Sierra Leone.

